# Microarray analysis of gene expression profiles of cardiac myocytes and fibroblasts after mechanical stress, ionising or ultraviolet radiation

**DOI:** 10.1186/1471-2164-6-6

**Published:** 2005-01-18

**Authors:** Marjan Boerma, Caroline GC van der Wees, Harry Vrieling, J Peter Svensson, Jan Wondergem, Arnoud van der Laarse, Leon HF Mullenders, Albert A van Zeeland

**Affiliations:** 1 Department of Toxicogenetics, Leiden University Medical Center, Wassenaarseweg 72, 2333 AL Leiden, The Netherlands; 2 Department of Cardiology, Leiden University Medical Center, Albinusdreef 2, 2333 ZA Leiden, The Netherlands; 3 Department of Clinical Oncology, Leiden University Medical Center, Albinusdreef 2, 2333 ZA Leiden, The Netherlands

## Abstract

**Background:**

During excessive pressure or volume overload, cardiac cells are subjected to increased mechanical stress (MS). We set out to investigate how the stress response of cardiac cells to MS can be compared to genotoxic stresses induced by DNA damaging agents. We chose for this purpose to use ionising radiation (IR), which during mediastinal radiotherapy can result in cardiac tissue remodelling and diminished heart function, and ultraviolet radiation (UV) that in contrast to IR induces high concentrations of DNA replication- and transcription-blocking lesions.

**Results:**

Cultures enriched for neonatal rat cardiac myocytes (CM) or fibroblasts were subjected to any one of the three stressors. Affymetrix microarrays, analysed with Linear Modelling on Probe Level, were used to determine gene expression patterns at 24 hours after (the start of) treatment. The numbers of differentially expressed genes after UV were considerably higher than after IR or MS. Remarkably, after all three stressors the predominant gene expression response in CM-enriched fractions was up-regulation, while in fibroblasts genes were more frequently down-regulated. To investigate the activation or repression of specific cellular pathways, genes present on the array were assigned to 25 groups, based on their biological function. As an example, in the group of cholesterol biosynthesis a significant proportion of genes was up-regulated in CM-enriched fractions after MS, but down-regulated after IR or UV.

**Conclusion:**

Gene expression responses after the types of cellular stress investigated (MS, IR or UV) have a high stressor and cell type specificity.

## Background

The mammalian myocardium contains several cell types, of which the cardiac myocytes (CM) make up most of the heart's mass. Although a small proportion of CM in the adult myocardium remains mitotic, most CM lose the capacity to undergo cell division shortly after birth [1]. In the adult heart, approximately 70% of the cells is represented by non-myocytes, most of which belong to the fibroblast compartment. In this study, we investigate how the stress response of cardiac cells to increased mechanical stress (MS) can be compared to genotoxic stresses induced by two DNA damaging agents, ionising radiation (IR) and ultraviolet radiation (UV).

In cardiac cells, MS is increased during excessive pressure or volume load of the heart, as seen in hypertensive and valvular heart disease. This results in an adaptive growth response leading to structural and functional cardiac changes, including CM hypertrophy and hyperplasia of fibroblasts, to compensate for the increased workload [2]. In vitro cyclic stretch of rat cardiac cells has been shown to be an appropriate model for cellular changes that occur during overload of cardiac muscle in vivo [3]. Mechanical signals may be transferred to the nucleus of cells through integrin receptors, cytoskeletal filaments and nuclear scaffolds [4] and through ion channels, ion exchangers and hormone receptors [5].

Radiation induced heart disease (RIHD) has been recognised as a late adverse effect of thoracic radiotherapy if the heart was situated in the radiation field [6]. IR induces the formation of reactive oxygen species that react with different components of the cell, thereby inducing macromolecular lesions. IR can activate several signal transduction pathways, involving growth factor receptors, death receptors and DNA damage sensing proteins [7]. Primary fibroblasts in culture are known to go into senescence shortly after IR [8], after which terminal differentiation of these cells is induced [9]. Cultures of CM do not demonstrate cell death, nor a loss of function upon a single dose of 10,000 rad (~100 Gy) [10,11].

The density of DNA damaging events after UVC, which include helix-distorting photolesions, is three orders of magnitude higher than the density of DNA damage that occurs after IR [12] and sufficient to block cellular DNA replication and transcription. Signal transduction after UV is mediated via components of the cellular membrane, involving growth factor receptors, and via DNA damage sensing proteins [7]. UV is known to induce cell cycle arrest and apoptosis in several cell types [13,14].

The aim of the study was to identify and compare differentially expressed genes (up-regulated or down-regulated when compared with untreated controls) in cardiac cells in response to MS, IR or UV. To this purpose, cultures enriched for ventricular CM or fibroblasts were exposed to one of the three stressors. Differentially expressed genes were identified using Affymetrix GeneChips. Several statistical methods have been developed to analyse Affymetrix gene expression microarrays. We used a method based on Linear Modelling on Probe Level [15] to describe the signal of every perfect match (PM) probe. Because overall changes in the expression of functionally related genes are more informative than the expression pattern of single genes, genes in microarray studies can be assigned to functional groups [16]. In the present study, such an approach was used to classify genes, based on biological function or on the role of a gene product in common intracellular pathways.

## Results

### Accuracy of the linear model

**Figure 1:**
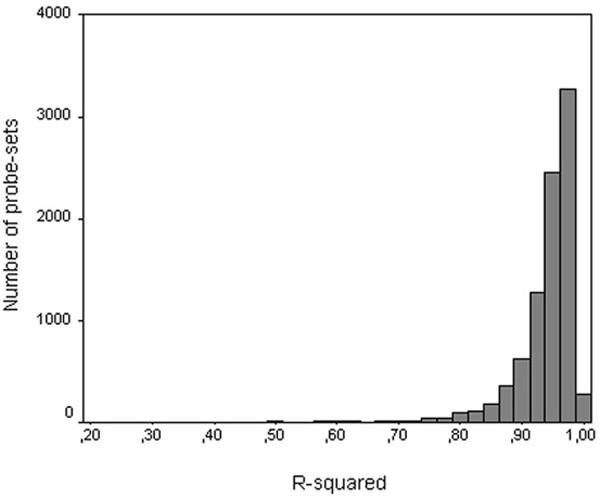
**Accuracy of the linear model**. Dot plot of all PM probe signals as calculated by the linear model, against actual PM probe signals determined from fibroblasts after UV (A). The data presented in Figure 1A were used to calculate correlation coefficients (R^2^) between the signals calculated by the linear model and the actual PM signals of fibroblasts after UV (B).

As an example for the accuracy of the linear model that was used to describe the PM probe signals, Figure 1A shows a dot plot of all PM probe signals as calculated by the linear model, against the actual PM probe signals determined from fibroblasts after UV. These data were used to calculate correlation coefficients (R^2^) between the signals calculated by the model and the actual PM signals obtained. Figure 1B shows the distribution of R^2 ^values for fibroblasts after UV. The majority of probe-sets have a correlation coefficient ≥ 0.90, indicating that the model used fitted the data accurately.

### Numbers of differentially expressed genes

**Figure 2:**
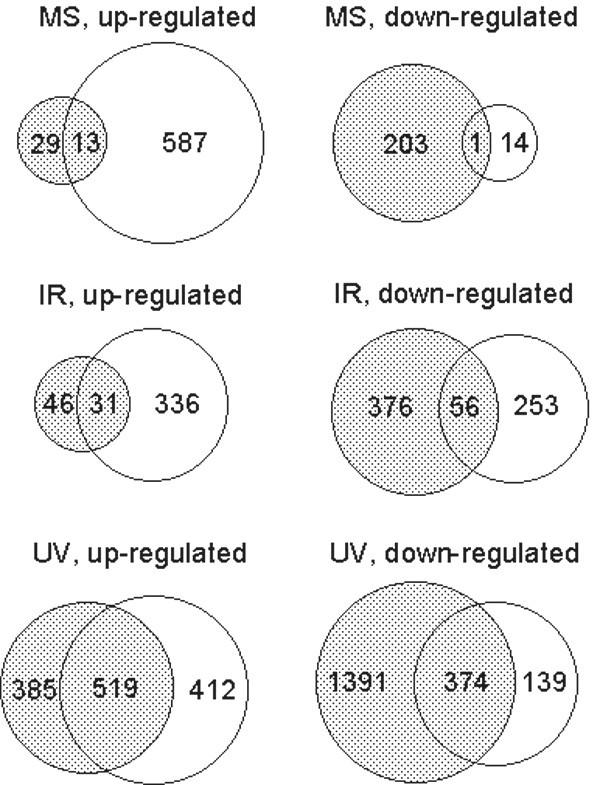
**Numbers of differentially expressed genes**. Numbers of differentially expressed genes (q < 0.005) with a unique LocusLink ID in cultures of fibroblasts (dotted) and CM-enriched cultures after MS (A), in cultures of fibroblasts (dotted) and CM-enriched fractions after IR (B), or in cultures of fibroblasts (dotted) and CM-enriched fractions after UV (C). Overlapping parts of the circles represent genes that show differential expression both in CM-enriched cultures/fractions and cultures of fibroblasts.

A Series entry (accession number GSE2032) at Gene Expression Omnibus (GEO), a public gene expression database of NCBI [17], gives access to all microarray data generated in this study. Figure 2 represents the numbers of genes with a unique LocusLink ID that were up-regulated or down-regulated (q < 0.005) in CM-enriched cultures/fractions and cultures of fibroblasts after one of the three stressors. When using these criteria, the numbers of differentially expressed genes (up-regulated or down-regulated) after UV were considerably higher than after IR or MS. After each of the three stressors more genes were up-regulated in CM-enriched cultures/fractions than in cultures of fibroblasts. Conversely, higher numbers of down-regulated genes were determined in fibroblasts. These differences were most pronounced after MS (Figure 2A).

**Figure 3:**
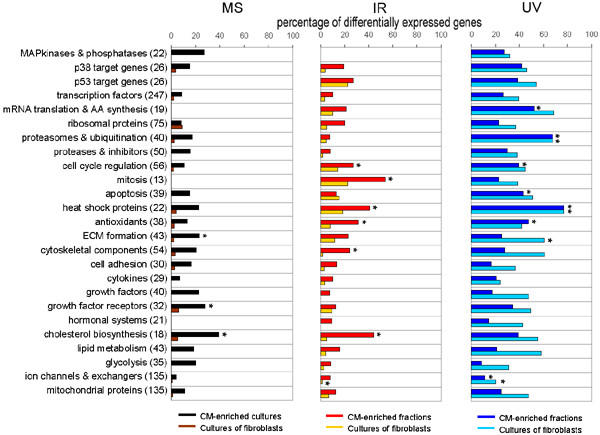
**Percentages of differentially expressed genes**. Percentage of total number of genes within functional groups that are differentially expressed in CM-enriched cultures/fractions and cultures of fibroblasts after MS, IR or UV. Numbers between brackets represent total numbers of genes within a functional group. For example, of the 22 MAPkinases and phosphatases found to be represented by the array, 27% were differentially expressed in CM-enriched fractions after UV. AA: amino acid. *Hypergeometric probability P < 0.005

Based on information available at NettAffx™ [18], extended with standard textbooks and recent literature, genes with a unique LocusLink ID were assigned to several functional groups. Subsequently, the status of each gene within a functional group was determined in both cell populations (CM-enriched cultures/fractions and cultures of fibroblasts). Individual probe-sets within these functional groups and their q-value after the three stressors are listed in table 1 (see additional file 1). In this table, down-regulated genes are distinguished from up-regulated genes by a minus-sign in front of their q-value. Figure 3 shows the percentage of genes in a functional group that are differentially expressed after MS, IR or UV. In accordance with Figure 2, the highest percentages of genes were differentially expressed after UV (on average 39.4% for both cell populations), followed by IR (13.0%). In general, the percentages of differentially expressed genes were lowest after MS (8.3%). Several functional groups, including heat shock proteins and genes involved in cholesterol biosynthesis, showed high proportions of differentially expressed genes with a hypergeometric probability P < 0.005. These functional groups were considered to have significantly high percentages of differentially expressed genes. On the other hand, in the group of genes encoding for ion channels and exchangers, low percentages of differentially expressed genes with a hypergeometric probability P < 0.005 were determined, both after IR and UV.

### Percentages of up-regulated genes

**Figure 4:**
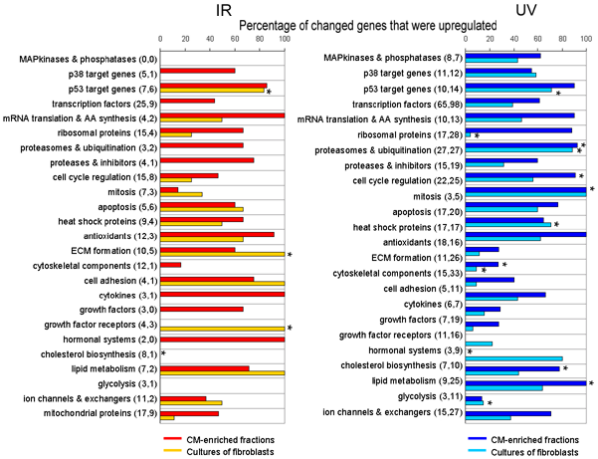
**Percentages of up-regulated genes**. Percentage of changed genes that were up-regulated per functional group after IR or UV. Numbers between brackets represent numbers of differentially expressed genes in CM-enriched fractions and cultures of fibroblasts, respectively. For example, of the 8 MAPkinases and phosphatases that were differentially expressed in CM-enriched fractions after UV, 63% were up-regulated. *Hypergeometric probability P < 0.005

Figure 4 shows the percentages of differentially expressed genes that were up-regulated after IR or after UV. In several functional groups, including p53 target genes and genes involved in mitosis, a significant percentage of differentially expressed genes was up-regulated (hypergeometric probability P < 0.005). Other functional groups, including cytoskeletal components and genes involved in cholesterol biosynthesis mainly had down-regulated genes. In all 25 functional groups, the hypergeometric probability P of the percentage of up-regulated genes after MS was not below the pre-set threshold of 0.005. Therefore, no significant percentages of up-regulated genes were determined in any of the functional groups after MS (data not shown).

### General stress response genes

Probe-sets that showed an up-regulation or down-regulation after more than one stressor are listed in table 2 (see additional file 2). The overlap in responsive genes between IR and UV was larger than the overlap between MS and one of the radiation types. Both after IR and UV, several genes that are known to play a central role in the radiation response of cells, including p21, GADD153 and mdm2, were up-regulated. These genes were not up-regulated after MS. A striking large proportion of genes encoding cytoskeletal components were down-regulated both after IR and UV.

### Validation of microarray results by semi-quantitative PCR

**Figure 5:**
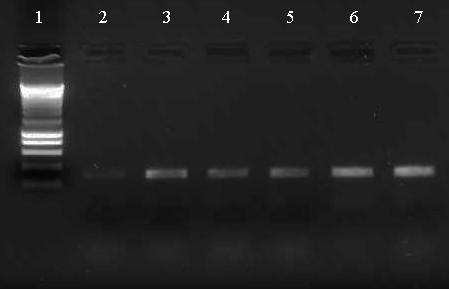
**Representative result of the Tenascin C PCR **Total RNA was isolated from CM-enriched cultures at 24 hours after MS or control treatment. After cDNA synthesis, semi-quantitative PCR was used to determine Tenascin C gene expression. Lane 1: smart ladder; lane 2: negative control; lane 3: positive control; lanes 4 and 5: CM-enriched after control treatment; lanes 6 and 7: CM-enriched after MS.

Gene expression changes detected using microarrays were validated by semi-quantitative PCR, using RNA from CM-enriched cultures at 24 hours after MS. The increased gene expression of Tenascin C and biglycan were confirmed with PCR in two independent experiments. In these two experiments, Tenascin C gene expression increased 1.59 and 1.64 times, respectively. Biglycan gene expression increased 1.42 and 1.25 times, respectively. Figure 5 shows a representative result of the Tenascin C PCR.

## Discussion

In this study, a linear model was used to describe GeneChip PM probe signals and to determine effects of three types of stressors on gene expression in two neonatal rat heart cell populations, consisting of CM that are known to be terminally differentiated cells and fibroblasts that still have the capacity to undergo mitosis. Several studies have shown a good correlation between Affymetrix GeneChip data and RT-PCR [16] or Northern-blot [19,20]. In a previous study neonatal rat CM-enriched cultures and cultures of neonatal rat cardiac fibroblasts were irradiated with a single dose of 8.5 Gy and some mRNA transcripts were quantified by competitive PCR [21]. In accordance with the present study, no significant changes in gene expression of transforming growth factor-β1 (TGF-β1), fibroblast growth factor-2 (FGF-2) and collagen type I were determined at 24 h after IR in cultures of cardiac fibroblasts. Moreover, no significant IR-induced changes were determined in gene expression of atrial natriuretic peptide (ANP) in CM-enriched cell populations in both the latter and the present study. On the other hand, CM-enriched cultures showed a reduced TGF-β1 expression (by PCR) at 24 h after 8.5 Gy, which was not observed in the present study. This might be due to differences in experimental design between the two studies, as in the former study CM-enriched cultures were obtained by incubation with bromodeoxyuridine to prevent fibroblast proliferation. Also, during and after irradiation, the cells were incubated in higher serum concentration than used in the present study.

Here, differences between the two cell populations are observed in the predominant type of gene expression response, i.e. up-regulation versus down-regulation. After all three stressors, differentially expressed genes were mostly up-regulated (q < 0.005) in CM-enriched fractions, while in cultures of fibroblasts the majority of changed genes were down-regulated. After MS, these differences were most pronounced. Paracrine signalling is involved in the response of CM and cardiac fibroblasts in co-cultures subjected to MS [22]. In the CM-enriched cultures used in this study, remaining fibroblasts might stimulate gene transcription of the CM, resulting in higher numbers of up-regulated genes. It cannot be excluded that differences in level of toxicity between the three stressors applied in this study caused differences in gene expression levels.

Of the three stressors examined in this study, both IR and UV are known to induce oxidative stress. Accordingly, both stressors induced high numbers of up-regulated genes involved in anti-oxidative processes. The high percentages of up-regulated genes encoding heat shock proteins in both cell populations after IR and UV might indicate oxidative stress induced protein damage in these cells. In comparison, after MS only few genes encoding heat shock proteins were up-regulated in either cell population.

Heinloth et al. (2003) proposed a model for the regulation of several gene expression patterns after IR and UV in human dermal fibroblasts, based on microarray analysis. The general view that p53 plays a central role in signal transduction after IR and UV was confirmed in their study. Moreover, IR down-regulated the expression of genes involved in mitosis. UV, on the other hand, induced the expression of genes involved in protein degradation and the MAPK pathway [23]. The latter data are in accordance with the data of the present study, showing that UV did affect genes participating in the MAPK pathway (although not significantly) in both cell populations, but IR did not. Moreover, in CM-enriched fractions, IR resulted in a down-regulation of a large percentage of genes involved in mitosis. Several genes, involved in cell cycle regulation and mitosis are expressed in foetal cardiac myocytes and down-regulated in adult myocytes, in accordance with their terminal differentiation [24,25]. Due to the close relation between foetal and neonatal cells, an expression of mitotic genes in the neonatal CM used in this study can be expected.

The high numbers of up-regulated genes involved in ubiquitination and protein degradation in both cell populations after UV are also in accordance with the study of Heinloth et al. (2003) and with other studies on cultured cells after UV [20]. These results might reflect the need of cells to replace molecules that were damaged by UV irradiation, although the proteasome is also proposed to play a role in several cellular processes, including DNA-repair, cell cycle regulation and cell survival after irradiation [26].

In CM-enriched cultures, MS led to a relatively high proportion of up-regulated genes that are involved in cholesterol biosynthesis. Among these genes, expression of 3-hydroxy-3-methylglutaryl-Coenzyme A reductase, which forms the starting enzyme of the mevalonate pathway and is considered to be the key enzyme in cholesterol biosynthesis, was up-regulated. This suggests that in neonatal rat cardiac myocytes cholesterol biosynthesis is stimulated after MS. Neonatal rat heart myocytes that undergo hypertrophy in culture show an increased biosynthesis and intracellular accumulation of cholesterol [27]. Moreover, the mevalonate pathway has been proposed to play a role in Ras activation in neonatal rat cardiac myocytes subjected to MS in culture, leading to hypertrophy [28]. *In vivo *hypertrophy of the heart is also associated with elevated myocardial cholesterol contents [29]. An increased cholesterol biosynthesis in cardiac myocytes that undergo MS might therefore accompany a hypertrophic response of these cells. However, of the three foetal genes that are known to be re-expressed in hypertrophic myocytes, i.e. smooth muscle alpha-actin, ANP and beta-myosin heavy chain, only the last gene was up-regulated at the time point of investigation. In contrast to MS, IR and UV led to a down-regulation of genes involved in cholesterol biosynthesis in CM-enriched fractions. In previous studies, alterations in cholesterol contents of cardiac myocytes and fibroblasts were associated with alterations in protein to DNA ratios, levels of several enzymes including ATPases and phosphatases, and diffusion rates of membrane proteins [30-32]. Moreover, a decrease in beating rate observed in aging cultures of CM was opposed by increased membrane amounts of cholesterol [33]. The role of cholesterol biosynthesis in the cellular functions of cardiac cells after MS, IR or UV needs further investigation.

Higher numbers of differentially expressed genes involved in ECM formation, including genes encoding collagens, fibronectin and laminin, were determined in CM-enriched cultures after MS than in cultures of fibroblasts. As mentioned before, paracrine signalling between CM and remaining fibroblasts might play a role in cellular responses in these CM-enriched cultures. Alterations in expression of genes involved in ECM formation might originate from remaining fibroblasts after stimulation by CM. Interestingly, MS does not affect the expression of these genes in cultures of fibroblasts, which suggests that paracrine signalling from CM is necessary for alterations in expression profiles of genes involved in ECM formation in fibroblasts.

The down-regulated genes encoding cytoskeletal components in cultures of fibroblasts after IR and UV, including myosin and troponin, are mostly CM-specific. Therefore, these genes are likely to originate from remaining CM in the fibroblast cultures, although their numbers were low (3–5%). The extremely low numbers of differentially expressed genes encoding for ion channels and exchangers might be explained by a low number of cardiac cell type-specific genes within this functional group.

## Conclusions

MS, IR and UV mainly induce stress-specific and cell-type specific gene expression profiles in neonatal rat CM-enriched cultures and cultures of neonatal rat heart fibroblasts. Functional groups that show significant percentages of differentially expressed genes suggest that certain cellular pathways are activated after one or more stresses.

## Methods

### Cell culturing

The experiments were performed with permission of the local committee on animal experiments, installed by the University of Leiden according to the Dutch law. Cardiac cells were isolated from ventricles of neonatal rat hearts, and cultured as described before [14,34]. By applying a pre-plating method, cultures enriched for CM and cultures of fibroblasts were obtained.

After 2 days of culturing with 5% horse serum (HS), culture media of CM-enriched cultures were replaced by media containing 2.5% HS. One day later, these cultures were subjected to UV, IR or MS.

Three days after isolation, cultures of fibroblasts were subcultured for 6 days in medium containing 10% foetal bovine serum (FBS). Then, culture medium was replaced by medium containing 2.5% FBS. One day later, confluent cell cultures were subjected to UV, IR or MS.

### Irradiation and cyclic stretch

Cells were subjected to a single dose of 8.5 Gy of X-rays at room temperature, using a 6 MV accelerator (SL 75-5 Philips), operated at a dose rate of 8 Gy min^-1^. During irradiation, sham-irradiated control cells were kept at room temperature. After irradiation, cell cultures were maintained at 37°C for 24 h.

In another experiment cells were irradiated with 10 J/m^2 ^UVC at room temperature at a dose rate of 0.2 J/m^2 ^per s. Before irradiation, medium was collected and cells were rinsed with PBS. Following irradiation, growth medium was returned to the cultures and the cells were incubated for an additional 24 h. Control cells received a similar treatment except for UV irradiation.

To subject cells to cyclic stretch, CM-enriched cells (54 ± 5% CM) and fibroblasts (95 ± 2% non-myocytes) were cultured in 6-well Flex I culture plates (Flexcell, Hillsborough, NC, USA), coated with collagen I. Medium was changed to medium containing 2.5% serum and after 24 h plates were placed in the Flexercell Strain Unit FX-2000^® ^(Flexcell) in which the frequency and magnitude of stretch were regulated by a computer-controlled vacuum pump. The apparatus applied an equiaxial cyclic stretch of 20% elongation to the wells at a frequency of 60 cycles/min (1 Hz). Stretch was applied for 24 h. Control cells were grown in identical culture plates and incubated in the same incubator as the stretched cultures, but were not mounted in the Flexercell Strain Unit.

### Cell harvesting after irradiation

Twenty-four hours after IR or UV, non-irradiated and irradiated CM-enriched cultures were trypsinised and subjected to centrifugal elutriation [35]. The proportion of CM in each elutriation fraction was determined by flow cytometric analysis of myosin expression, as described before [35]. After elutriation of IR-exposed cultures, CM-enriched fractions contained 74 ± 3% CM and after elutriation of UV-exposed cultures, CM-enriched fractions contained 85 ± 3% CM. Because of the high purity of the fibroblast cultures (95 ± 2% non-myocytes), no centrifugal elutriation was applied on these cells. CM-enriched fractions and cultures of fibroblasts were used for RNA isolation.

### Cell harvesting after MS

After undergoing MS or control treatment for 24 h, cells were collected from CM-enriched cultures and from cultures of fibroblasts by trypsinisation. The proportion of CM in the cell cultures was determined by flow cytometric analysis of myosin expression as described before [35]. CM-enriched cell cultures, containing 54 ± 5% CM, and fibroblast cultures, containing 95 ± 2% non-myocytes, were used for RNA isolation.

### RNA isolation and labelling

RNA was isolated applying an RNeasy^® ^kit (Qiagen GmbH, Hilden, Germany), according to the manufacturer's instructions. Ten to 14 μg of total RNA from CM-enriched fractions and 7.5 to 14 μg of total RNA from fibroblast cultures was used for labelling. Per experiment, the quantities of total RNA from irradiated and control cells were equal. cDNA was synthesised using the Gibco BRL Superscript system (Invitrogen, Carlsbad CA, USA). Briefly, single stranded cDNA was synthesised using Superscript II reverse transcriptase and T7-oligo(dT)24 primers at 42°C for 1 h. Double stranded cDNA was obtained by using DNA ligase, DNA polymerase I and RNAse H at 16°C for 2 h, followed by T4 DNA polymerase at 16°C for 5 min. After clean up with a phase lock gel (Qiagen), ds-cDNA was used for *in vitro *transcription (IVT). cDNA was transcribed using the BioArray HighYield^® ^RNA transcript labelling kit (Enzo Lifesciences, Pharmingdale NY, USA) in the presence of biotin-labelled ribonucleotides, or using the MEGAScript T7^® ^kit (Ambion, Austin TX, USA), in the presence of biotin-labelled CTP and UTP. In each experiment, cDNAs from control cells and stressed cells were labelled simultaneously using the same labelling kit. After clean up with a RNeasy kit (Qiagen), the biotin-labelled IVT-RNA was fragmented in a buffer containing 40 mM Tris-acetate (pH 8.1), 100 mM potassium-acetate and 30 mM magnesium-acetate, at 94°C for 35 min.

### Hybridisation of IVT-RNA

RG-U34A arrays (Affymetrix, Santa Clara CA, USA) were used, representing ~7000 transcripts, of which 6399 had a unique LocusLink identification number (ID) at the time of data analysis. Biotin-labelled IVT-RNA was hybridised to the arrays at 45°C for 16 h according to the manufacturer's instructions. After hybridisation, the arrays were washed in a GeneChip Fluidics Station 400 with a non-stringent wash buffer at 25°C followed by a stringent wash buffer at 50°C. After washing, the arrays were stained with a streptavidin-phycoerythrin complex. After staining, intensities were determined with a GeneChip scanner, controlled by GeneChip software (Affymetrix). The intensities were background corrected using gcrma [36] and normalized at the probe level by VSN [37].

### Data analysis

For each stressor, three independent experiments were performed. To describe the signal of every PM probe, the following linear model was used: signal ~P + T + E + TE, based on a method described before [15]. In our model, the symbols P, T, E and TE represent the effects of probe, treatment, experiment per stressor (1, 2, or 3) and the interaction between treatment and experiment, respectively. Subsequently, analysis of variance was applied on the treatment effect to determine the p-value for each probe-set. The p-values were corrected for multiple testing using the Benjamini Hochberg step-up procedure [38] which yielded q-values for the false discovery rates (FDR). The FDR level of control was set to 0.005, equivalent to selecting genes with a q-value<0.005 (keeping up- and down-regulated genes separate by the sign of the treatment coefficient). Some genes are represented by more than one probe-set. A gene was determined to be differentially expressed when at least 50% of the probe-sets representing this gene showed significant up-regulation or down-regulation.

To determine significant proportions of differentially expressed genes within functional groups, the hypergeometric probability P was calculated. P < 0.005 was considered significant.

To determine the accuracy of the linear model that was used to describe PM signals, R^2 ^was calculated for every probe-set, using the following standard formula: R^2 ^= 1-ΣR_i_^2^/Σ(signal_i_-mean signal)^2^, where R_i _= observed-fitted value for signal_i _and mean signal = the mean of observed signal_i_.

To compare data on differentially expressed genes with microarray data in literature on species other than rat, RESOURCERER of the Institute of Genomic Research [39] was used.

### RNA isolation and semi-quantitative PCR

Total RNA isolation, cDNA synthesis and semi-quantitative PCR were performed as described before [40]. In short, total RNA was isolated using an RNeasy^® ^kit (Qiagen). Following DNAse (Amersham Pharmacia Biotech, Uppsala, Sweden) treatment, cDNA was synthesised using M-MLV reverse transcriptase (Life Technologies, Rockville, MD, USA) and oligo(dT) primers (Amersham). Semi-quantitative PCR was performed, using the following primers and annealing temperatures: Tenascin C sense: CGA CAG TTT TGT TAT CAG GAT CAG, Tenascin C antisense: GGC ACA TAA GTA ATC CGG AAA T, 60°C; Biglycan sense: CAA CAA CCC TGT GCC CTA CT, Biglycan antisense: GGT GT GCT TCT TTG CTT CC, 65°C. A competitive PCR was performed on GAPDH to correct for differences in cDNA concentrations. After agarose gel electrophoresis, intensities of PCR product bands were determined by Scion image analysis software (Scion Corporation, Frederick, MD, USA).

## Authors' contributions

MB performed experiments, developed and analyzed functional groups of genes and prepared the manuscript. CGCW performed experiments, developed and analyzed functional groups of genes and participated in discussions. HV and AL participated in discussions and gave textual advice. PS performed microarray normalization and data analysis. JW and LHFM participated in discussions. AAZ participated in discussions, suggested the assignment of genes to functional groups and gave textual advice. All authors read and approved the final manuscript.

## Supplementary Material

Additional File 1 **q-values of genes within functional groups **Table 1. Excel-file containing the 25 functional groups of genes used in this study. Of every gene, Affymetrix probe-set IDs and q-values after the three types of stress are listed. Down-regulated genes are represented by a minus-sign in front of their q-value. q-values < 0.005 are marked red (in case of up-regulation) and green (in case of down-regulation). Click here for file

Additional File 2 **General stress response genes **Table 2. Excel file containing Affymetrix probe-set IDs, LocusLink ID and gene description of all probe-sets that showed an up-regulation or down-regulation after two or more stresses. Click here for file
